# The major horse satellite DNA family is associated with centromere competence

**DOI:** 10.1186/s13039-016-0242-z

**Published:** 2016-04-27

**Authors:** Federico Cerutti, Riccardo Gamba, Alice Mazzagatti, Francesca M. Piras, Eleonora Cappelletti, Elisa Belloni, Solomon G. Nergadze, Elena Raimondi, Elena Giulotto

**Affiliations:** Dipartimento di Biologia e Biotecnologie, Università di Pavia, Via Ferrata 1, 27100 Pavia, Italy

**Keywords:** Horse genome, Centromere, Satellite DNA, Next generation sequencing, High resolution cytogenetics

## Abstract

**Background:**

The centromere is the specialized locus required for correct chromosome segregation during cell division. The DNA of most eukaryotic centromeres is composed of extended arrays of tandem repeats (satellite DNA). In the horse, we previously showed that, although the centromere of chromosome 11 is completely devoid of tandem repeat arrays, all other centromeres are characterized by the presence of satellite DNA. We isolated three horse satellite DNA sequences (37cen, 2P1 and EC137) and described their chromosomal localization in four species of the genus Equus.

**Results:**

In the work presented here, using the ChIP-seq methodology, we showed that, in the horse, the 37cen satellite binds CENP-A, the centromere-specific histone-H3 variant. The 37cen sequence bound by CENP-A is GC-rich with 221 bp units organized in a head-to-tail fashion. The physical interaction of CENP-A with 37cen was confirmed through slot blot experiments. Immuno-FISH on stretched chromosomes and chromatin fibres demonstrated that the extension of satellite DNA stretches is variable and is not related to the organization of CENP-A binding domains. Finally, we proved that the centromeric satellite 37cen is transcriptionally active.

**Conclusions:**

Our data offer new insights into the organization of horse centromeres. Although three different satellite DNA families are cytogenetically located at centromeres, only the 37cen family is associated to the centromeric function. Moreover, similarly to other species, CENP-A binding domains are variable in size. The transcriptional competence of the 37cen satellite that we observed adds new evidence to the hypothesis that centromeric transcripts may be required for centromere function.

**Electronic supplementary material:**

The online version of this article (doi:10.1186/s13039-016-0242-z) contains supplementary material, which is available to authorized users.

## Background

In mammals, a significant fraction of the genome is constituted by extended stretches of tandemly repeated DNA. It was shown that these highly repetitive sequences can give rise to satellite bands in gradient centrifugation experiments when they have a different GC content compared to bulk genomic DNA [1]; therefore, they were defined “satellite” DNA. In most eukaryotic chromosomes, these non-coding sequences are the main DNA component of centromeric and pericentromeric heterochromatin [2–6].

Although the centromeric function is highly conserved through eukaryotes, centromeric satellite DNA is rapidly evolving, often being species specific [6–8]. Moreover, following our initial description of a centromere completely devoid of satellite DNA in the horse [9], other examples of naturally occurring satellite-less centromeres were observed in plants and animals [10–13]. These observations raise the challenging question whether centromeric and pericentromeric satellites have a functional role. A number of hypotheses have been proposed to explain the recruitment, by the majority of eukaryotic centromeres, of large stretches of satellite DNA. Satellite DNA may facilitate binding of the centromere specific histone CENP-A (the main epigenetic mark of centromere function) to centromeric chromatin [14]. In addition, centromeric repetitive DNA, typically devoid of active genes, may aid the formation of a heterochromatic environment which would favour the stability of the chromosome during mitosis and meiosis [6, 7, 15]. In several species, centromeric satellite DNA is transcribed and it has been suggested that these transcripts may play a role in heterochromatin formation. Transcription of the centromeric regions seems to be important for chromatin opening and CENP-A loading; these transcripts are believed to provide a flexible scaffold that allows assembly or stabilization of the kinetochore proteins and may act *in trans* on all or on a subset of chromosomes, independently of the primary DNA sequence [16–18].

In a previous work, we isolated two horse satellites, 37cen and 2PI, from a genomic library in lambda phage [19], and investigated their chromosomal distribution in four equid species [10]. More recently [20], we described a new horse satellite, EC137, which is less abundant than 37cen and 2PI and mostly pericentromeric. In the horse, 37cen, 2PI and EC137 are present, together or individually, at all primary constrictions, with the exception of the centromere of chromosome 11 which is completely satellite-free [9, 10, 21]. In this work, we applied next-generation DNA sequencing and high-resolution cytogenetic approaches to identify the satellite repeat bearing the centromeric function in the horse and we proved that this satellite is transcriptionally active.

## Results and discussion

### Molecular identification of the functional centromeric satellite DNA

The aim of the present work was to define the satellite DNA repeats bearing the centromeric function in the horse. To this purpose, an anti-CENP-A antibody [9, 21] was used in immunoprecipitation experiments with chromatin from horse skin primary fibroblasts. DNA purified from immunoprecipitated and from control non-immunoprecipitated chromatin (input) was paired-end sequenced through an Illumina HiSeq 2000 platform. A total of 78,207,302 and 41,155,660 high-quality reads were obtained from ChIP and input samples, respectively. It is important to remind that most mammalian centromeres are not assembled due to their highly repetitive nature and that all mammalian genome data bases include a “virtual” chromosome, named “unplaced”, composed of contigs containing highly repetitive DNA sequences (a number of which are located at the centromeres) that lack chromosome assignment. Therefore, in the EquCab2.0 reference genome, we expected to identify most of the centromeric repeats binding CENP-A in “unplaced” contigs. Each contig is identified by a number which is unrelated to its genomic location.

Sequence reads were aligned through Bowtie 2.0 [22] to the horse reference genome (EquCab2.0, 2007 release). Peak-calling was performed with the default parameters of MACS 2.0.10 software [23] using the input reads as control dataset and applying stringent criteria (see Materials and Methods) to select significantly enriched regions [24]. A total of 1705 regions mapping on 1462 unplaced contigs were significantly enriched, as shown in Additional file 1: Table S1.

The sequence of the 1705 enriched regions was downloaded from the nucleotide database [25] and compared, with the MultAlin software [26], to all known equine repetitive elements, retrieved from the Repbase database [27, 28]; 97 % (1653/1705) of these repetitive fragments consisted of the 37cen satellite (SAT_EC at [28]). In all these regions the 37cen 221 bp units were organized in a head-to-tail fashion.

**Fig. 1:**
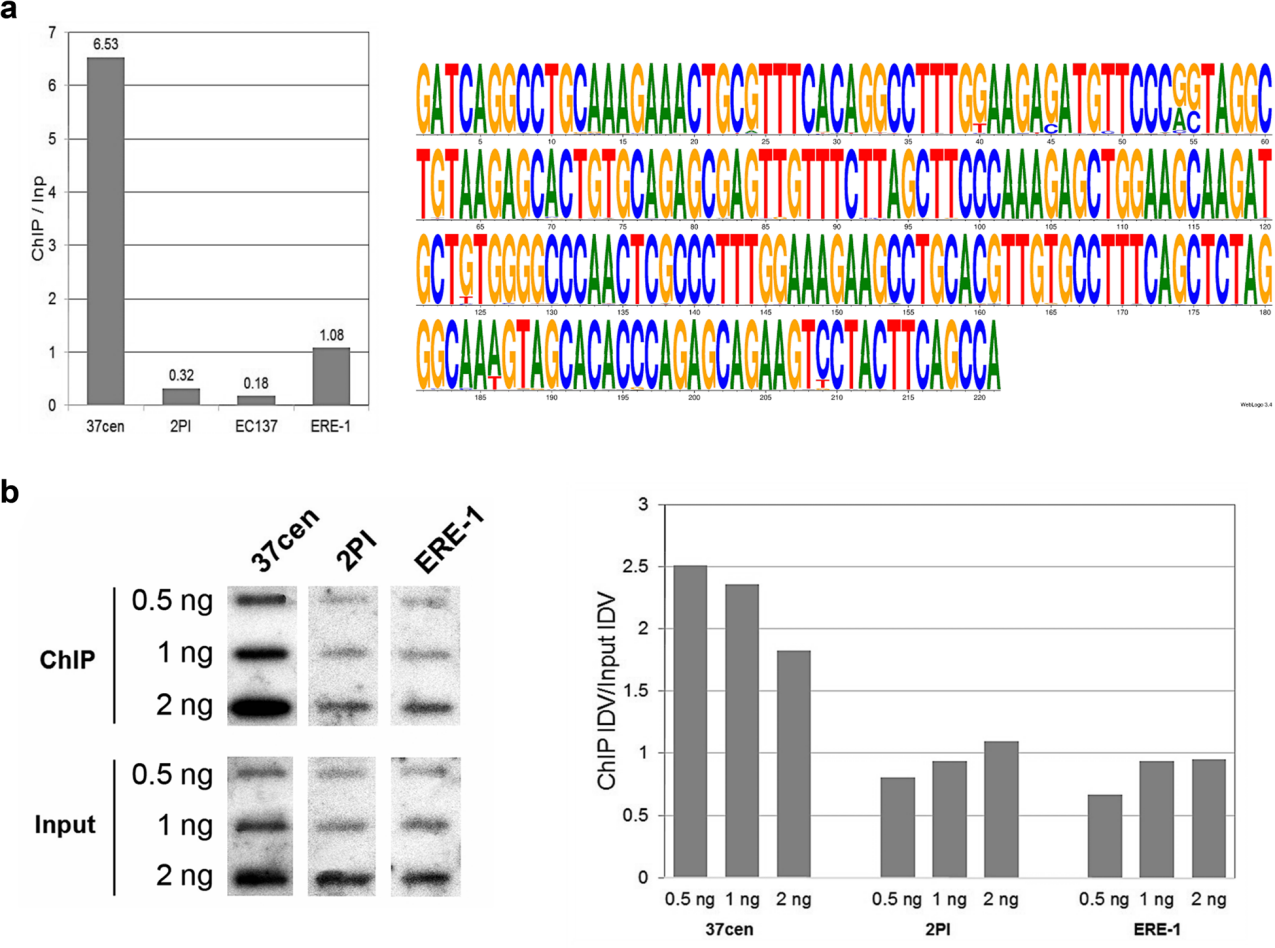
Identification and analysis of the CENP-A bound horse satellite. **a**: In the left panel, the enrichment of the 37cen, 2P1 and EC37 satellites was measured as ratio between normalized read counts in immunoprecipitated and in input DNA. The same calculation was performed for the ERE1 retrotransposon sequence. The right panel shows the 221 bp consensus sequence of the CENP-A bound 37cen satellite. **b**: Slot-blot analysis. Left panel: hybridization of P^32^ labelled probes (37cen, 2PI and ERE-1) with DNA purified from chromatin immunoprecipitated with CENP-a (*top*) and from non immunoprecipitated chromatin (*bottom*). Right panel: densitometric analysis of slot-blot hybridizations

We then aligned the reads from input and from immunoprecipitated chromatin with the consensus sequence of 37cen (SAT_EC at [28]), of the pericentromeric satellite 2PI (SAT2pl at [28]) and of the ERE-1 retrotransposon, that is interspersed throughout the genome (ERE1 at [28]); we also aligned them with the sequence of the pericentromeric satellite EC137 (GenBank JX026961, [20]). The alignment was performed using the Razers3 software [29] allowing 20 % of mismatches. The number of reads was normalised to take into account the total number of reads in each sample and the length of the consensus sequence; raw read counts are reported in Additional file 2: Table S2. To quantify the enrichment of these sequences in CENP-A bound chromatin, we calculated the ratio between normalized read counts in the immunoprecipitated and in the input DNA (Fig. 1a, left panel). A 6.5-fold enrichment was observed for the 37cen satellite; 2PI and EC137 were under-represented in the immunoprecipitated chromatin, while ERE1 was equally represented in the two fractions. These results demonstrate that 37cen is the main functional centromeric satellite sequence.

To better define the sequence actually bound by CENP-A, we deduced a consensus from the 33,902,776 reads mapping on the 37cen reference (Additional file 2: Table S2). The consensus is shown as logo in Fig. 1a right panel. Although 20 % of mismatches were allowed in selecting the 37cen reads, the newly defined consensus is very similar to the previously reported consensus suggesting that 37cen units are highly conserved both in CENP-A bound and unbound DNA.

AT richness has been considered a typical feature of centromeric chromatin [30], however, this idea has been recently a subject of debate [8]. The GC content of 37cen is 53 % thus confirming that GC richness is compatible with the centromeric function.

To further confirm the association of the 37cen satellite DNA with centromeric function, horse chromatin was immunoprecipitated with the anti-CENP-A antibody [9, 21]. Purified immunoprecipitated and input DNA was blotted and hybridized with probes for 37cen, 2PI and ERE-1 repeats (Fig. 1b). The results showed that the 37cen hybridization signal was more intense in immunoprecipitated than in input DNA; conversely, the signal intensity obtained after hybridization with the 2PI and ERE-1 probes was comparable or even lower in immunoprecipitated than in input DNA blots. The Integrated Densitometric Value (IDV) of signals was calculated with the ImageJ 1.48v software [31]. As reported in Fig. 1b, right panel, the ratio between immunoprecipitated and input values for 37cen was comprised between 1.8 and 2.5 confirming that this satellite is enriched in CENP-A bound chromatin. On the opposite, no enrichment of 2PI and ERE-1 repeats was observed.

These results demonstrate that, although at horse centromeric and pericentromeric regions the different satellite families form a complex mosaic of intermingled segments [20], only the 37cen family is involved in the centromeric function. This situation is similar to that previously described in other species, such as humans, where alpha satellite only is bound by CENP-A whereas other satellite families seems to play an accessory function [6].

### Transcription of the 37cen satellite

**Fig. 2:**
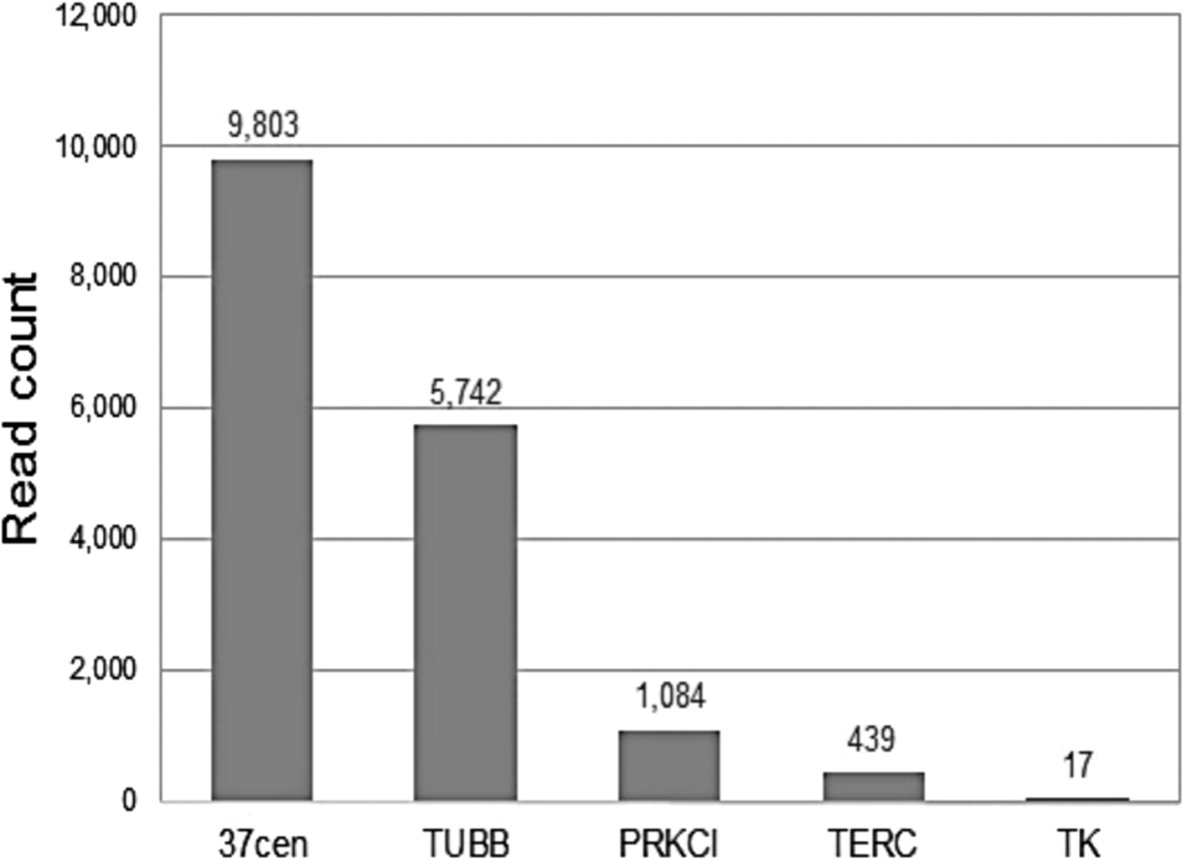
Transcription of the 37cen satellite by RNA-seq. The graph reports the number of reads corresponding to the consensus sequences of 37cen, tubulin beta (TUBB), protein kinase C iota (PRKCI), telomerase RNA component (TERC), thymidine kinase (TK)

A large body of evidence demonstrates that centromeric and pericentromeric satellite DNA is transcribed in a number of species from yeast to mammals [18]. We analysed, by means of RNA-seq, the transcriptome profile of a horse fibroblast cell line in order to search for 37cen transcripts. Out of the 59,090,294 RNA-seq reads analysed, we detected 9803 reads corresponding to the consensus sequences of 37cen (Fig. 2). The alignment with a 37cen dimer was performed using the Razers3 software [29] and allowing 20 % of mismatches. We also counted the number of reads corresponding to 442 nt long transcripts from four genes: *TUBB (tubulin beta), PRKCI (protein kinase C iota), TERC (telomerase RNA component), TK (thymidine kinase)* (Fig. 2). The results show that the number of 37cen reads is comparable or higher than that observed for the analysed genes.

From these data we cannot infer the transcription level of single 37cen units nor the fraction of transcriptionally active units. It has been suggested that centromeric transcripts may have an impact on development, cell differentiation, and response to environmental stimuli [4, 6] and it is generally agreed that transcription competence is a prerequisite for centromere functioning and kinetochore assembly [32–34]. Emerging evidence suggests that satellite transcripts may act both *in cis* and *in trans* [5, 35]. Therefore, in the horse system, it is tempting to speculate that 37cen RNA may play a role not only at satellite-based centromeres but also at the satellite-less centromere of chromosome 11.

### High resolution cytogenetic analysis

**Fig. 3:**
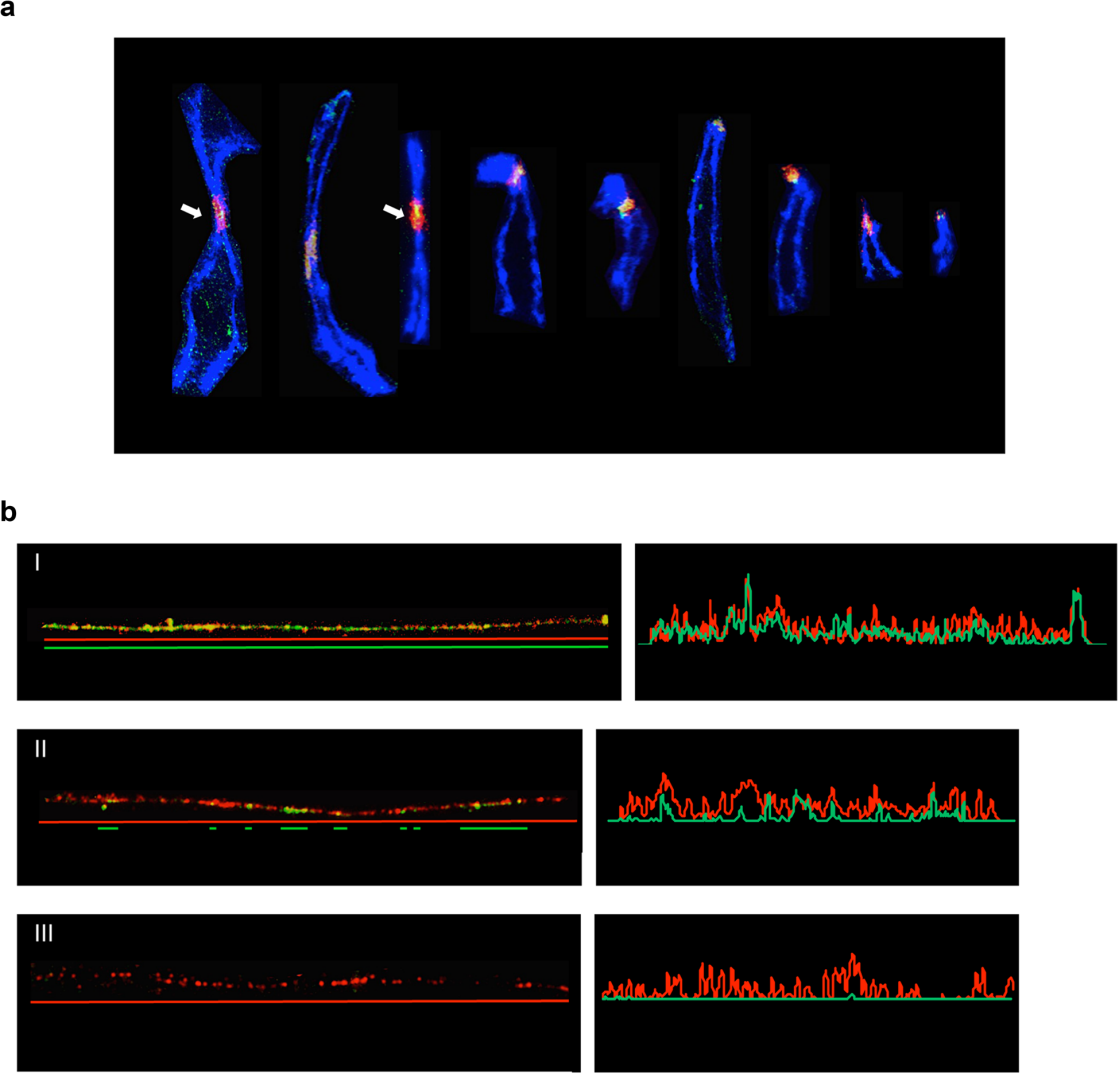
**a**: Immuno-FISH on mechanically stretched chromosomes. 37cen is red labelled while CENP-A, detected by a CENP-A enriched CREST serum is green labelled. A total number of 99 stretched chromosomes was analysed. A sample of representative images is reported in the figure. **b**: Immuno-FISH on extended chromatin fibres. The 37cen satellite DNA is labelled in red. CENP-A, identified with a CENP-A enriched CREST serum is green labelled. In each panel, under the microscope image of the fibre, the CENP-A binding pattern observed is sketched. Images on the right show line graphs quantifying the fluorescence staining along the length of each fibre. **I**: CENP-A covers the whole length of the 37cen positive region. **II**: CENP-A binding regions are arranged in blocks of variable length intermingled in the 37cen positive stretch. **III**: A chromatin fibre with no CENP-A binding is reported

Our previous FISH analyses, on stretched chromosomes and combed DNA fibres, demonstrated that horse centromeric and pericentromeric regions display a mosaic arrangement of different satellite DNA families [20]. To analyse the physical organization of the centromeric domains, we carried out immuno-FISH experiments on mechanically stretched chromosomes using 37cen as FISH probe (red in Fig. 3a) and a previously tested [21] CREST serum (green in Fig. 3a) to mark the centromeric domain. A total number of 99 stretched chromosomes (46 meta- or submeta-centric and 53 acrocentric) was examined, a representative panel of which is shown in Fig. 3a. Although the results of this type of experiments can only be considered semi-quantitative, the abundance of the 37cen sequence appeared highly variable among chromosomes, extending in some instances over a large pericentromeric region (white arrows in Fig. 3a) or being apparently confined to the primary constriction. As expected, the CREST signals always colocalized with the 37cen fluorescence, however, no clear correlation seemed to exist between intensity and extension of the 37cen and the CREST signals.

These results suggest that, at horse centromeres, the size of CENP-A binding domains is not related to the extent of satellite DNA stretches; these finding are in agreement with the well described inter- and intra-specific variability of the molecular organization of eukaryotic centromeres [6].

To define more precisely the relationship between 37cen and the centromeric function, a higher-resolution immuno-FISH analysis was performed on horse chromatin fibres. A total number of 25 extended fibres was analysed, some representative examples of which are reported in Fig. 3b. Different arrangements of CENP-A domains were observed: although 60 % of the fibres (15/25) showed CENP-A binding covering the whole length of the 37cen positive region (I in Fig. 3b), in 28 % (7/25) of the cases (II in Fig. 3b) CENP-A domains appeared as blocks of variable length intermingled into 37cen stretches. The observation of the discontinuous presence of CENP-A at centromeres resembles the chromatin organization observed using the same high resolution morphological approach in human cells and in *Drosophila* [36]*.* Our ChIP results (see Fig. 1a) demonstrated that only a fraction of all genomic 37cen repeats is associated with centromere function; the detection of the FISH signal without CENP-A binding (III in Fig. 3b) on 12 % (3/25) of the fibres further confirmed this result; this fraction of fibres may derive from pericentromeric locations, that were shown to contain the 37cen satellite by our analysis on stretched chromosomes (Fig. 3a).

## Conclusions

The primary constriction of mammalian chromosomes is typically embedded in a constitutive heterochromatic environment characterized by long arrays of tandemly repeated satellite DNA. Centromeric satellite repeats are extremely variable in length and composition, not only between and within species but also among chromosomes of the same individual [7]. The horse is peculiar among mammalian species because the centromere of chromosome 11 is completely devoid of satellite DNA [9, 10, 21]. Satellite-based horse centromeres are constituted by the two major classes of equid satellite DNA, 37cen and 2PI, flanked by the pericentromeric accessory satellite EC137 [20]. In the present paper, we proved that only the GC rich 37cen sequence is associated with the centromeric function and is transcriptionally active. We also showed that the horse shares with other species a similar molecular organization of centromeres, relying on CENP-A blocks of variable length immersed in long satellite DNA stretches [36].

The significance of satellite DNA at mammalian centromeres has so far been elusive because satellite-less centromeres are perfectly functional [9, 21]. In the horse, the presence of satellite-based together with a satellite-less centromere makes this species a particularly suitable model for future studies on the role of centromeric tandem repeats.

## Methods

### Ethics statement

Horse DNA, RNA, chromosomes and chromatin samples were obtained from previously established primary fibroblast cell lines [21]. These cell lines were established from skin samples taken from animals not specifically sacrificed for this study; the animals were being processed as part of the normal work of the abattoirs.

### Cell lines

Horse skin primary fibroblasts were were cultured in DMEM medium (EuroClone) supplemented with 20 % foetal bovine serum, 2 mM L-glutamine, 1 % penicillin/streptomycin and 2 % non-essential amino acids at 37 °C with 5 % CO_2_. Cytogenetic analysis demonstrated that the cell lines had a diploid modal chromosome number (2n = 64) and a normal karyotype.

### Chromatin Immuno-Precipitation (ChIP) and sequencing (ChIP-seq)

Chromatin was prepared from horse primary fibroblasts, following cross-linking with 1 % formaldehyde and sonication. Immunoprecipitation was performed using a purified CENP-A polyclonal [9, 21], raised against the N-terminus of human CENP-A, kindly provided by Prof. Mariano Rocchi (University of Bari). The immunocomplex was purified using A/G beads (nProtein A Sepharose™ 4 Fast Flow/Protein G Sepharose™ 4 Fast Flow, GE Healthcare). After reverse cross-linking, carried out overnight at 65 °C, immunoprecipitated and input DNAs were extracted with the “Wizard Genomic DNA Purification Kit” (Promega) according to the manufacturer’s instructions.

Immunoprecipitated and input DNAs were then paired-end sequenced through an Illumina HiSeq2000 platform by IGA Technology Services [37]. Sequence reads were aligned to the horse reference genome (EquCab2.0, 2007 release) with Bowtie 2.0 [22] and peak-calling was performed through the software MACS version 2.0.10 20120605 [23], using default parameters. Stringent criteria [24] were applied to identify significantly enriched regions: fold enrichment > 5, pile-up > 100, -log_10_(p-value) > 100, -log_10_(q-value) > 100.

To quantify the number of reads corresponding to each repetitive element, the reads from immunoprecipitated DNA and input DNA were mapped to a reference constituted by the consensus sequences of 37 cen (“SAT_EC” on repbase, [27, 28]), 2PI (“SAT2pl” on repbase), ERE-1 (“ERE1” on repbase) and EC137 (GenBank JX026961). The alignment was performed with the Razers3 software [29] using all of the reads from the paired-end sequencing as a whole single-end dataset; the mapping was carried out using default parameters with exception of percent identity threshold (-i option) which was set to 80. For each sequence type analysed, read counts from immunoprecipitated and input DNA were calculated with the “SAM/BAM to Counts 1.0.0” tool, available on the Galaxy platform [38]. Each read count value was normalized with respect to the total number of reads and to the length of the reference sequence. To measure enrichment due to immunoprecipitation with CENP-A, the ratio between normalized read counts in the immunoprecipitated and input samples was calculated.

### Slot-blot analysis

DNA purified from chromatin imunoprecipitated with the anti CENP-A antibody [9, 21] and input DNA were transferred to nylon membranes (Amersham HybondTM-N, GE Healthcare) through a Minifold II apparatus (Schleicher and Schuell) and denatured. The membranes were hybridized at 64 °C for 18 h in Church buffer containing one of the following ^32^P-α[dCTP]-labelled probes, generated by random primer labelling: a 7 kb EcoRI/SacI 37cen fragment and a 7.2 kb EcoRI/SacI 2PI fragment [10]; a 441 bp PCR-amplified fragment from horse genomic DNA, containing an ERE-1 insertion [39].

After hybridization, the membranes were washed twice in 2× SSC, 0.5 % SDS for 15 min at 64 °C and once in 0.2× SSC, 0.5 % SDS for 30 min at 64 °C. Radioactive signals were detected using a phosphorimager (Cyclone, Packard) and the densitometric analysis was performed with the ImageJ 1.48v software [31].

### RNA extraction and sequencing (RNA-seq)

RNA extraction from whole cells was performed using QIAzol Lysis Reagent (QIAGEN) according to the manufacturer’s instructions. To eliminate DNA contaminations, RNA was treated twice with RNase-free DNase-I (Promega), and then purified with the RNA Clean and Concentration kit (ZYMO Research). After library preparation using Illumina TruSeq Stranded Total RNA with Ribo-Zero GOLD, the resulting cDNA was paired-end sequenced by IGA Technology Services [37] through an Illumina HiSeq2000 platform.

RNA-seq reads were mapped, with the same Razers3 parameters as the ChIP and input datasets, on a reference composed of a dimer of the 37cen consensus sequence (“SAT_EC” on repbase) and on 442 bp long portions of the following transcripts: TUBB (XM_001491178.5, nucleotides 488 to 929), PRKCI (XM_014732748.1, nucleotides 605 to 1046), TERC (NR_001566.1 nucleotides 9 to 450), TK (XM_001491081.5 nucleotides 26 to 467). The same length was used for each sequence in order to have comparable read counts without normalization.

### Immuno-FISH

Mechanically stretched chromosomes and extended chromatin fibres were prepared as previously described [20, 21]. Immunofluorescence was carried out using a CENP-A enriched CREST serum [21] for CENP-A detection, and a plasmid containing the 37cen satellite as FISH probe [20]; immuno-FISH experiments on stretched chromosomes and chromatin fibres were carried out as previously described [21]. Digital grey-scale images were acquired with a fluorescence microscope (Zeiss Axioplan) equipped with a cooled CCD camera (Photometrics). Pseudocoloring and merging of images were performed using the IpLab software (Scanalytics Inc.). For fluorescence quantification of 37cen (red signal) and CENP-A (green signal) on chromatin fibres, separate channel digital images were converted in text images using ImageJ 1.48v [31]. The mean fluorescence intensity of each antibody spot was calculated point by point along the fibre length and plotted in a line chart.

## Additional files

Additional file 1: Table S1. Enriched regions found on the unplaced contigs. The columns represent: the accession number of the contigs, the start and end position of the enriched region within the contig, the length of the region, and the statistical parameters calculated by the peak caller [pile-up, fold enrichment, -log10(p-value), -log10(q-value)]. Regions are listed according to their contig number. (XLS 211 kb) Additional file 2: Table S2. un-normalized read counts from ChIP-seq experiment and input control. (XLS 27 kb)

## Abbreviations

CENP-A: centromere protein A
ChIP: chromatin immunoprecipitation
ChIP-seq: ChIP sequencing
CREST: calcinosis, Raynaud’s phenomenon, esophageal dysmotility, sclerodactyly, and telangiectasia
ERE-1: equine repetitive element 1
FISH: fluorescence *in situ* hybridization
IDV: integrated densitometric value
MACS: model-based analysis of ChIP-seq
PRKCI: protein kinase C iota
RNA-seq: RNA sequencing
TERC: telomerase RNA component
TK: thymidine kinase
TUBB: tubulin beta
